# Multi-omics approaches reveal that diffuse midline gliomas present altered DNA replication and are susceptible to replication stress therapy

**DOI:** 10.1186/s13059-024-03460-y

**Published:** 2024-12-20

**Authors:** Anastasia E. Hains, Kashish Chetal, Tsunetoshi Nakatani, Joana G. Marques, Andreas Ettinger, Carlos A. O. Biagi Junior, Adriana Gonzalez-Sandoval, Renjitha Pillai, Mariella G. Filbin, Maria-Elena Torres-Padilla, Ruslan I. Sadreyev, Capucine Van Rechem

**Affiliations:** 1 Department of Pathology, Stanford University, Stanford, CA 94305 USA; 2 Department of Molecular Biology, Massachusetts General Hospital, Harvard Medical School, Boston, MA 02114 USA; 3 Department of Pathology, Massachusetts General Hospitaland, Harvard Medical School , Boston, MA 02114 USA; 4 Institute of Epigenetics and Stem Cells, Helmholtz Munich, Munich, Germany; 5 Department of Pediatric Oncology, Dana-Farber Boston Children’s Cancer and Blood Disorders Center, Boston, MA 02215 USA; 6 Broad Institute of Harvard and MIT, Cambridge, MA 02142 USA

**Keywords:** Diffuse midline gliomas H3 K27-altered, H3K27M, Cell cycle, Replication timing, Replication stress

## Abstract

**Background:**

The fatal diffuse midline gliomas (DMG) are characterized by an undruggable H3K27M mutation in H3.1 or H3.3. K27M impairs normal development by stalling differentiation. The identification of targetable pathways remains very poorly explored. Toward this goal, we undertake a multi-omics approach to evaluate replication timing profiles, transcriptomics, and cell cycle features in DMG cells from both H3.1K27M and H3.3K27M subgroups and perform a comparative, integrative data analysis with healthy brain tissue.

**Results:**

DMG cells present differential replication timing in each subgroup, which, in turn, correlates with significant differential gene expression. Differentially expressed genes in S phase are involved in various pathways related to DNA replication. We detect increased expression of DNA replication genes earlier in the cell cycle in DMG cell lines compared to normal brain cells. Furthermore, the distance between origins of replication in DMG cells is smaller than in normal brain cells and their fork speed is slower, a read-out of replication stress. Consistent with these findings, DMG tumors present high replication stress signatures in comparison to normal brain cells. Finally, DMG cells are specifically sensitive to replication stress therapy.

**Conclusions:**

This whole genome multi-omics approach provides insights into the cell cycle regulation of DMG via the H3K27M mutations and establishes a pharmacologic vulnerability in DNA replication, which resolves a potentially novel therapeutic strategy for this non-curable disease.

**Supplementary Information:**

The online version contains supplementary material available at 10.1186/s13059-024-03460-y.

## Background

The temporal control of DNA replication, i.e., replication timing, is highly regulated. This is necessary to ensure that the genome is entirely and correctly duplicated during S phase. Replication timing is associated with the epigenetic landscape [1], gene expression [2], and cell fate [3]. Replication timing is highly dysregulated in diseases, including cancers [4, 5]. Alteration of the progression of the replication fork leads to replication stress, DNA damage, and associates with genomic instability [6, 7]. Replication stress creates vulnerabilities that can be therapeutically targeted in cancers [8]. Despite these observations, the relationship between specific cancer alterations, replication timing, and associated cellular processes remains largely unexplored.

Replication timing exhibits distinct features: initiation zones (origin of replication from which the replication forks diverge in opposite directions), timing transition regions (replication forks moving away from initiation sites), constant timing regions (early and late plateaus of replication), and termination sites (replication forks converging and terminating replication) [9]. Replication timing and its different features associate with distinct epigenetic patterns [1]. One such example is H3K27me3, which is enriched in regions replicating during mid to late S phase and associates with timing transition regions and termination sites [1]. The H3K27me3 landscape is reshaped in many cancers due to alterations in the chromatin modifiers writing or erasing this mark or in the histone H3 itself [10].

Diffuse midline gliomas (DMG), H3 K27-altered, are non-curable brain cancers primarily diagnosed in children. More than 80% of DMG feature a K27M mutation in either *HIST1H3B/C*, coding for the replicative histone H3.1, or *H3F3A*, coding for the variant H3.3 [11, 12]. These mutations define two DMG subgroups presenting differences in phenotypes, response to therapy, and prognosis [13]. Despite these differences, commonalities and dissimilarities between these subgroups remain largely unexplored outside of gene regulation.

While the H3.1 replicative histone is inserted in the entire genome as the replication fork progresses, the histone variant H3.3 is systematically deposited where it was before DNA replication, displacing nascent H3.1 and creating boundaries between H3.3 and H3.1 [14]. These H3.1/H3.3 boundaries demarcate the initiation zones of the early origins of replication [14]. In *Drosophila*, the genomic deposition of H3.1K27M and H3.3K27M is consistent with the DNA replication-dependent deposition of H3.1 and the predominantly replication-independent deposition of H3.3 [15]. In *C. elegans*, incorporation of the H3.3K27M mutation in one of the five H3.3 genes leads to germline defects: ectopic activation of DNA replication, accumulation of DNA damage, and aberrant progression of the cell cycle [16]. Corroborating the importance of cell cycle progression, oncohistones need to be incorporated in the chromatin via cell cycling for the K27M mutations to propagate a pathogenic effect [15]. While the relationship between oncohistones, cell cycle, and DNA replication was demonstrated in model organisms, replication timing within the DMG subgroups, its association with gene expression, their impact on measurable features of replication stress, and potential associated therapeutic vulnerabilities have yet to be described.

In this study, we leverage whole genome multi-omics approaches to evaluate DMG cells (H3.1K27M and H3.3K27M subgroups), normal astrocytes, tumors, and normal brain cells for novel cellular control that allows therapeutic vulnerabilities to be established for targetable pathways in DMG. Our studies resolved a critical role for H3K27M in replication timing genome-wide, while establishing an opportunity to therapeutically intervene with replication stress therapies.

## Results

### H3.1K27M DMG, H3.3K27M DMG, and normal astrocytes harbor differential DNA replication timing

**Fig. 1:**
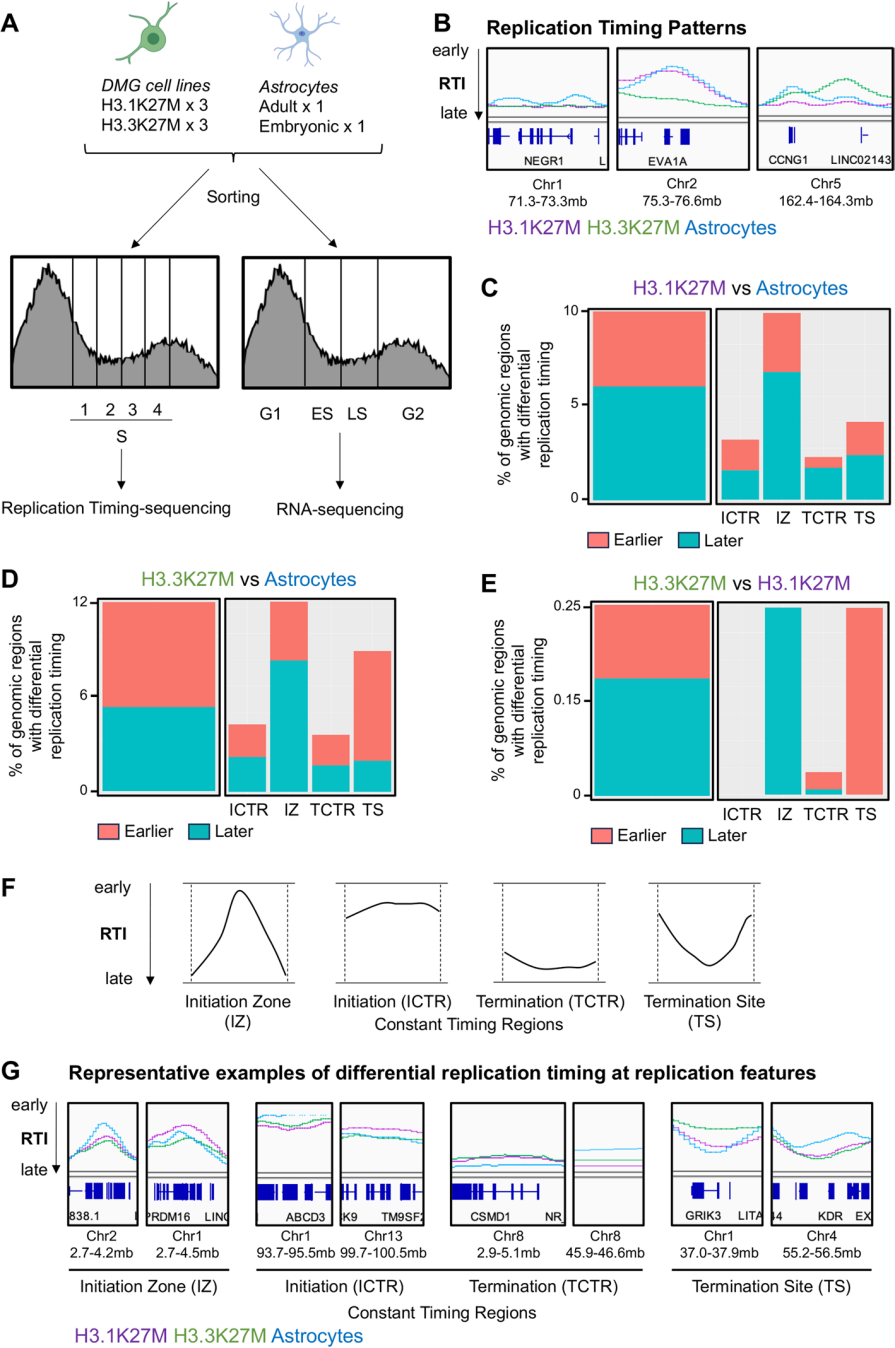
H3K27-altered DMG feature differential replication timing with H3.1K27M/H3.3K27M-subgroup specificities.** A** Schematic of the experimental setup. Three DMG cell lines of each subgroup (H3.1K27M and H3.3K27M) and two normal astrocyte cell lines were used. For replication timing sequencing S phase was divided into four equal cell populations. For cell-cycle RNA sequencing cells were divided into four groups consisting of G1, early S (ES), late S (LS), and G2/M. **B** Representative genomic tracks at the regions with differential replication timing between cell types (H3.1K27M in purple, H3.3K27M in green, astrocytes in blue), RTI = replication timing index (from top to bottom, replication earlier to later during S phase). Each line represents an average of biological replicates. **C**–**E** Differential replication timing of H3.1K27M (**C**) and H3.3K27M (**D**) compared to astrocytes and of H3.3K27M compared to H3.1K27M (**E**) (earlier and later replication represented in coral and teal, respectively). Left panels: percentage of genomic regions presenting differential replication timing between respective cell types. Right panels: differential replication timing at specific replication features. H3.1K27M/Astrocytes: 2224 regions replicating earlier and 3379 regions replicating later; H3.3K27M/Astrocytes: 3780 regions replicating earlier and 3038 regions replicating later; H3.3K27M/H3.1K27M: 60 regions replicating earlier and 96 regions replicating later. **F** Schematic representation of replication features: IZ = initiation zones, TCTR = termination constant timing regions, ICTR = initiation constant timing regions, TS = termination sites. **G** Representative examples of genomic regions with differential replication timing at replication features

To evaluate DMG H3 K27-altered in the context of the cell cycle, we explored replication timing throughout the S phase and gene expression profiles over the entire cell cycle in DMG and non-cancer brain cells. We leveraged a Fluorescence Activated Cell Sorting (FACS)-based method for these studies to avoid chemical-induced alterations and remove such confounders in the assessment [1]. With the goal of identifying new therapeutic opportunities that would not affect non-cancer cells in the same environment, we used three patient-derived pediatric DMG cell lines harboring the H3.1K27M mutation (DIPG4, DIPG21, DIPG33), three DMG cell lines harboring the H3.3K27M mutation (DIPG19, DIPG24, DIPG29), and two normal astrocyte cell lines (NHA, HA-bs). Oligodendrocyte precursor cells, the likely cell of origin of DMG [17], have a very low doubling capacity (2 weeks), which precludes their use for cell cycle-centric studies. However, astrocytes are abundant and have a comparable proliferative capacity in the normal brain, making them ideal to compare to the DMG cells. Each of these primary cell lines was sorted based on DNA content in four equal phases across S phase (S1, S2, S3, S4) for replication timing sequencing [1, 18], and in four phases across the entire cell cycle (G1, early S (ES), late S (LS), G2/M) for RNA sequencing [1] (Fig. 1A).

Replication timing sequencing characterizes the time during S phase at which specific genomic regions duplicate. Cells are pulse-labeled with BrdU and FACS sorted in four phases across S phase based on their DNA content. The BrdU-labeled DNA is then immunoprecipitated and prepared for sequencing [1, 18, 19]. This method maps nascent DNA across S phase, therefore determining which regions of the genome replicate “early” or “late” during the DNA replication process. This information is translated into a replication timing index (RTI): quantification of replication timing at a given genomic region as one continuous value between 0 and 1, the strongest read density in early (S1) and late (S4) phase resulting in RTI = 0.10 or 0.91, respectively (Fig. 1B) [1, 20].

Using this approach, we observed that H3.1K27M, H3.3K27M, and astrocytes presented differential replication timing patterns at various genomic loci (Fig. 1B and Additional file 1: Fig. S1). Some regions presented differential replication timing for astrocytes (Fig. 1B, left panel), while others presented differential replication timing for only one subgroup of DMG (Fig. 1B, middle panel), or both DMG subgroups and astrocytes presented unique patterns (Fig. 1B, right panel). Overall, H3.1K27M and H3.3K27M presented differential replication timing of 10% and 12% of the genome when compared to astrocytes, respectively (Fig. 1C–D). Over half of the regions presenting differential timing replicated later in H3.1K27M DMG than in astrocytes (2224 regions replicating earlier and 3379 regions replicating later) (Fig. 1C, left panel), whereas over half of the regions presenting differential timing replicated earlier in H3.3K27M DMG than in astrocytes (3780 regions replicating earlier and 3038 regions replicating later) (Fig. 1D, left panel). While comparing directly H3.3K27M to H3.1K27M presented a much higher variability, which is anticipated from cell lines established post-mortem from patients that underwent different treatment regimens, these genotypes presented differential replication timing of 0.25% of the genome (60 regions replicating earlier and 96 regions replicating later) (Fig. 1E).

DNA replication exhibits local replication features (Fig. 1F–G). These defined features include initiation zones (IZ, clusters of origin of replication where the replication forks diverge in opposite directions from the same point, Fig. 1F left panel), constant timing regions (ICTR and TCTR, early and late plateaus of replication, Fig. 1F middle panels), and termination sites (TS, replication forks converging and terminating replication, Fig. 1F right panel) [9]. In comparison to normal astrocytes, H3.1K27M cell lines presented genomic regions exhibiting local DNA replication features replicating earlier or later, with a higher proportion of differential replication at initiation zones (Fig. 1C, right panel). H3.3K27M cell lines also presented genomic regions exhibiting local DNA replication features replicating earlier or later, but with a higher proportion of differential replication at initiation zones and termination sites (Fig. 1D, right panel). H3.1K27M and H3.3K27M differed mainly at initiation zones and termination sites, with H3.3K27M cell lines presenting later replication at initiation zones and earlier replication at termination sites (Fig. 1E). These results indicate that H3.1K27M DMG cell lines, H3.3K27M DMG cell lines, and astrocyte cell lines present distinct replication timing profiles.

### Differential replication timing correlates with differential gene expression

**Fig. 2:**
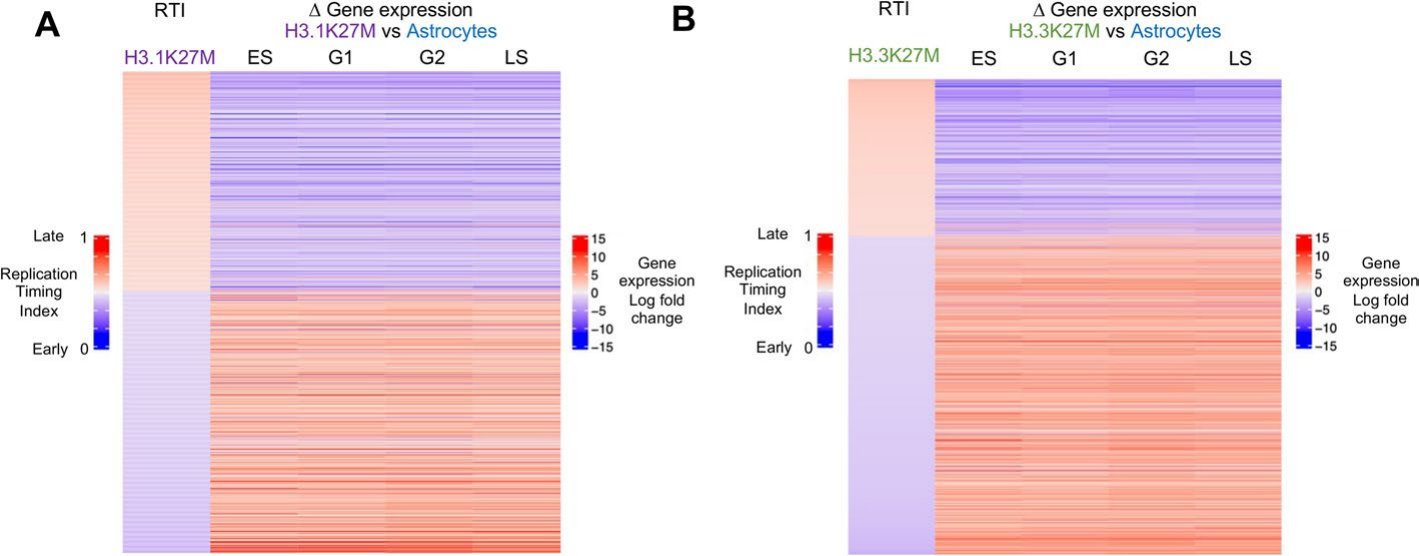
Differential replication timing correlates with differential gene expression within the same genomic regions.** A**–**B** Heatmap correlating the replication timing index (RTI, the value between 0 for early and 1 for late replication) with gene expression changes in the same regions (downregulated and upregulated in blue and red, respectively) for the genomic regions presenting differential replication timing between H3.1K27M DMG cell lines and astrocytes (872 regions, **A**) and between H3.3K27M and astrocytes (1369 regions, **B**)

We then assessed whether altered replication timing was connected to differential gene expression in DMG. Regions presenting differential replication timing between DMG cell lines and astrocytes were compared to the differential gene expression within these regions in any given cell cycle phase (Fig. 2A–B, Additional file 1: Fig. S2A, Additional file 2: Tables S1–S3). Regions replicating later in DMG cell lines (RTI > 0.5, red) were associated with downregulated gene expression (log fold change < 0, blue) and vice versa (Fig. 2A–B). This correlation was also observed between DMG subgroups (Additional file 1: Fig. S2A). These regions were enriched in initiation zones when comparing H3.1K27M to astrocytes (Additional file 1: Fig. S2B), in initiation zones and termination sites when comparing H3.3K27M to astrocytes (Additional file 1: Fig. S2C), and in termination sites when comparing H3.3K27M to H3.1K27M (Additional file 1: Fig. S2D). These data are consistent with studies linking earlier replication timing to gene expression and later replication timing with gene suppression [2].

### H3.1K27M and H3.3K27M DMG exhibit changes in gene expression in all phases of the cell cycle when compared to normal astrocytes

**Fig. 3:**
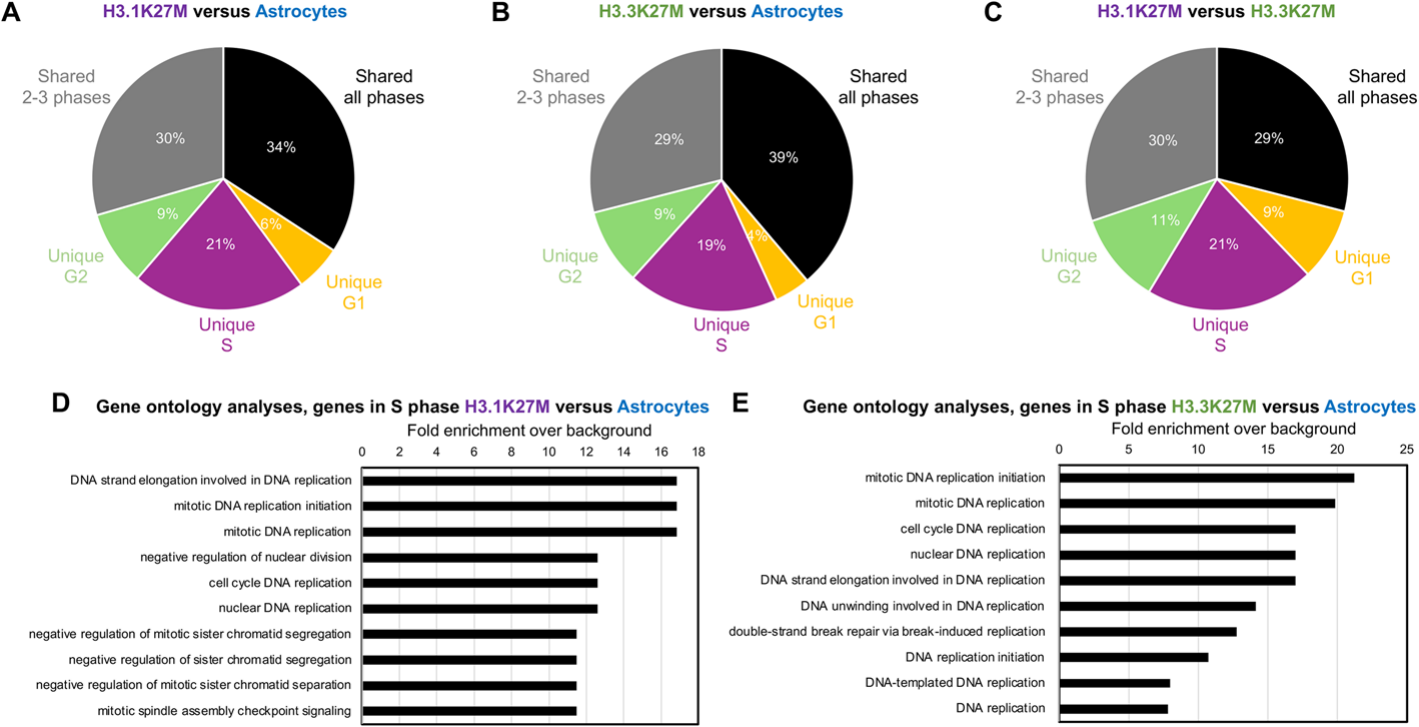
H3K27-altered DMG differentially express DNA replication-related pathways in S phase. **A-C** Pie charts representing the percentages of genes differentially expressed uniquely in G1, S, or G2, shared between 2 and 3 cell cycle phases, or shared across all phases between H3.1K27M DMG cell lines and astrocytes (**A**), between H3.3K27M DMG cell lines and astrocytes (**B**), and between H3.1K27M and H3.3K27M cell lines (**C**). **D**–**E** Gene Ontology analysis of the biological processes for differentially expressed genes in S phase in H3.1K27M compared to astrocytes (**D**) and in H3.3K27M compared to astrocytes (**E**). Fold enrichment = over-representation over background

Given the patterns of differential gene expression and replication timing, we further investigated gene expression in different cell cycle phases (G1, ES, LS, G2/M) in H3.1K27M DMG cell lines, H3.3K27M DMG cell lines, and astrocytes (Fig. 1A). When comparing DMG subgroups and astrocytes, we identified genes exhibiting differential expression specifically in each cell cycle phase and genes with differential expression across several phases (Fig. 3A–C and Additional file 1: Fig. S3A–C). Although most of the genes with differential expression were shared among all cell cycle phases (34% for H3.1K27M versus astrocytes (Fig. 3A and Additional file 1: Fig. S3A), 39% for H3.3K27M versus astrocytes (Fig. 3B and Additional file 1: Fig. S3B), and 29% for H3.1K27M versus H3.3K27M (Fig. 3C and Additional file 1: Fig. S3C)), many genes presented differential gene expression uniquely in S phase (21% for H3.1K27M versus astrocytes (Fig. 3A and Additional file 1: Fig. S3A), 19% for H3.3K27M versus astrocytes (Fig. 3B and Additional file 1: Fig. S3B), and 21% for H3.1K27M versus H3.3K27M (Fig. 3C and Additional file 1: Fig. S3C)).

Gene ontology analyses [21–23] of genes differentially expressed in S phase between DMG cell lines and astrocytes revealed enrichment in DNA replication-related pathways in both H3.1K27M and H3.3K27M (Fig. 3D–E). While the number of differentially expressed genes in S phase between H3.1K27M and H3.3K27M subgroups were too small for statistically significant results after gene ontology analyses (420 genes, Additional file 1: Fig. S3C), the top two PANTHER pathways were threonine biosynthesis and salvage pyrimidine deoxyribonucleotides (data not shown). Of note, thymidine kinase 1, an enzyme regulating intracellular thymidine pool and tightly regulated across the cell cycle [24, 25], presented significantly higher expression in H3.1K27M specifically in the early S phase.

### H3.1K27M and H3.3K27M DMG exhibit altered cell cycle regulation of DNA replication genes

**Fig. 4:**
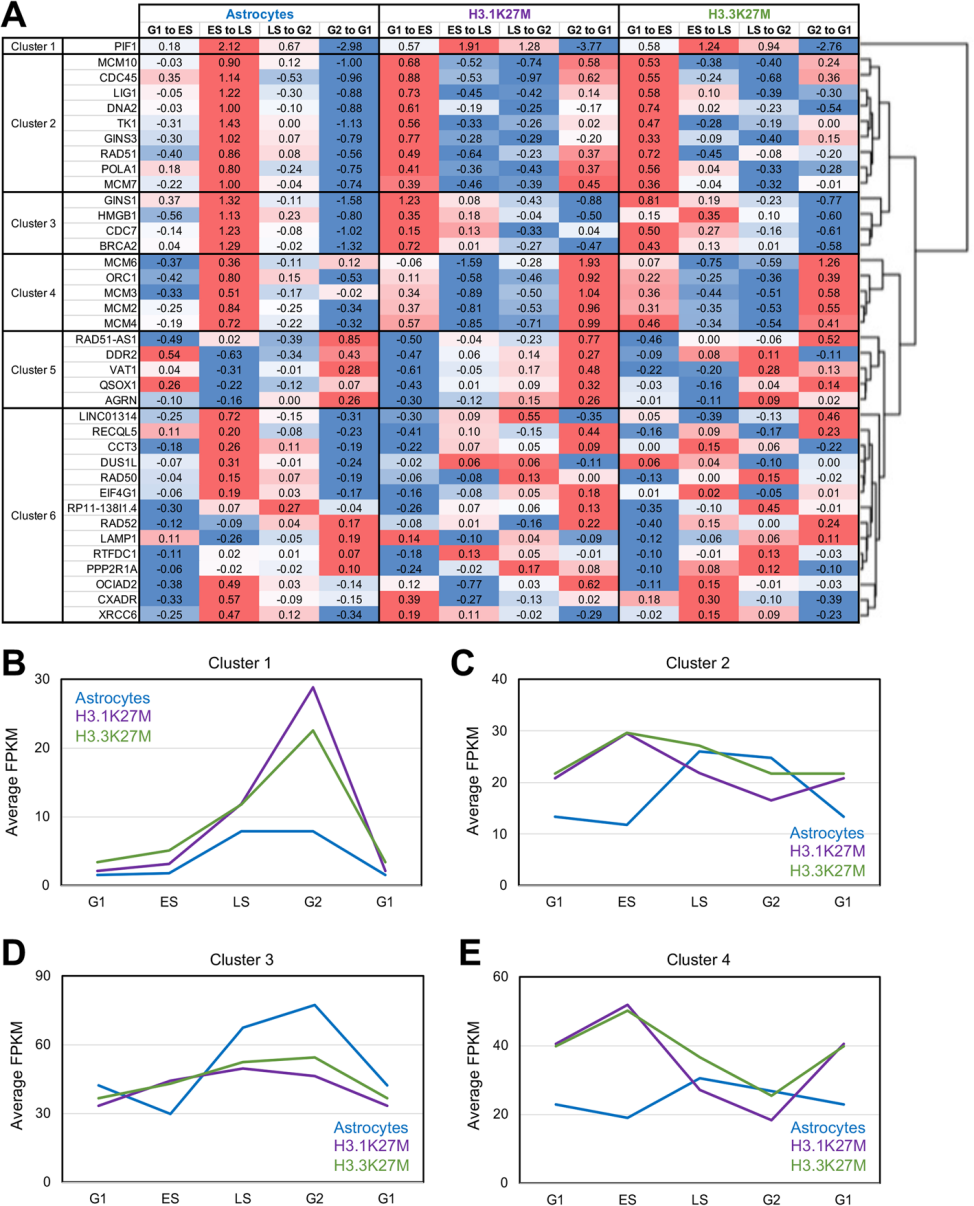
H3.1K27M and H3.3K27M DMG exhibit altered cell cycle regulation of DNA replication genes.** A** Heatmap representing the clustering of DNA replication genes (from PANTHER) according to their differential expression in subsequent phases of the cell cycle in astrocytes, H3.1K27M, and H3.3K27M DMG cell lines. Numbers represent the ratio of expression in one phase compared to the previous one (G1 to ES: expression in ES divided by expression in G1, etc.). Color: scale of blue to red with blue = downregulated from one phase to the next phase and red = upregulated from one phase to the next phase. **B**–**E** Graphs representing the average of FPKM (fragments per kilobase per million mapped fragments) of genes from cluster 1 (**B**), cluster 2 (**C**), cluster 3 (**D**), and cluster 4 (**E**) for each phase of the cell cycle and each subgroup (astrocytes in blue, H3.1K27M in purple, H3.3K27M in green)

Because genes involved in DNA replication-related pathways were differentially expressed between DMG cell lines and normal astrocytes in S phase, we took the list of 39 genes under the DNA replication category of PANTHER [23] to assess their expression across the cell cycle in our cell lines. For this, we computed the ratio of expression between each consequent phase in the cell cycle (G1 to ES, ES to LS, LS to G2, G2 to G1) and applied hierarchical clustering to the gene expression ratios [26]. From this analysis, we observed six major clusters across 38 DNA replication genes (one gene was removed from the results because of its lack of expression in all cell lines used, Fig. 4A).

Clusters one, two, three, and four contained genes directly involved in DNA replication such as the replication complex itself and the homologous recombination DNA repair machinery involved in replication fork preservation [27]. These clusters presented the most variation of expression across the cell cycle. The only gene in cluster one, *PIF1*, encoding an RNA helicase at replication forks [28], started to be upregulated between G1 and ES in DMG cells and continued to be until G2. In contrast, it was only upregulated between ES and LS in normal astrocytes (Fig. 4A–B). While genes in clusters two and three were upregulated between ES and LS in astrocytes and downregulated between G2 and G1, these genes were upregulated earlier in DMG cells, between G1 and ES (Fig. 4A, C–D). Of note, some of these genes even started to be upregulated between G2 and G1 in H3.1K27M DMG specifically (cluster two, Fig. 4A). In astrocytes, genes in cluster four were upregulated at the same time as the ones in clusters two and three (between ES and LS), but they were upregulated much earlier in DMG cells, between G2 and G1 (Fig. 4A, E).

Clusters five and six contained genes more indirectly involved in DNA replication and presented a more stable expression across the cell cycle in astrocytes and DMG subgroups (Fig. 4A and Additional file 1: Fig. S4A–B). Of note, genes in cluster five were overall less expressed in H3.3K27M DMG in comparison to astrocytes and H3.1K27M DMG (Additional file 1: Fig. S4A), while genes in cluster six were less expressed in both DMG subgroups in comparison to astrocytes (Additional file 1: Fig. S4B).

Together, DNA replication-related genes were differentially regulated across the cell cycle in DMG H3 K27-altered compared to astrocytes, with commonalities and dissimilarities between DMG subgroups.

### DMG cell lines present shorter inter-origin distance and slower replication forks than normal astrocytes

**Fig. 5:**
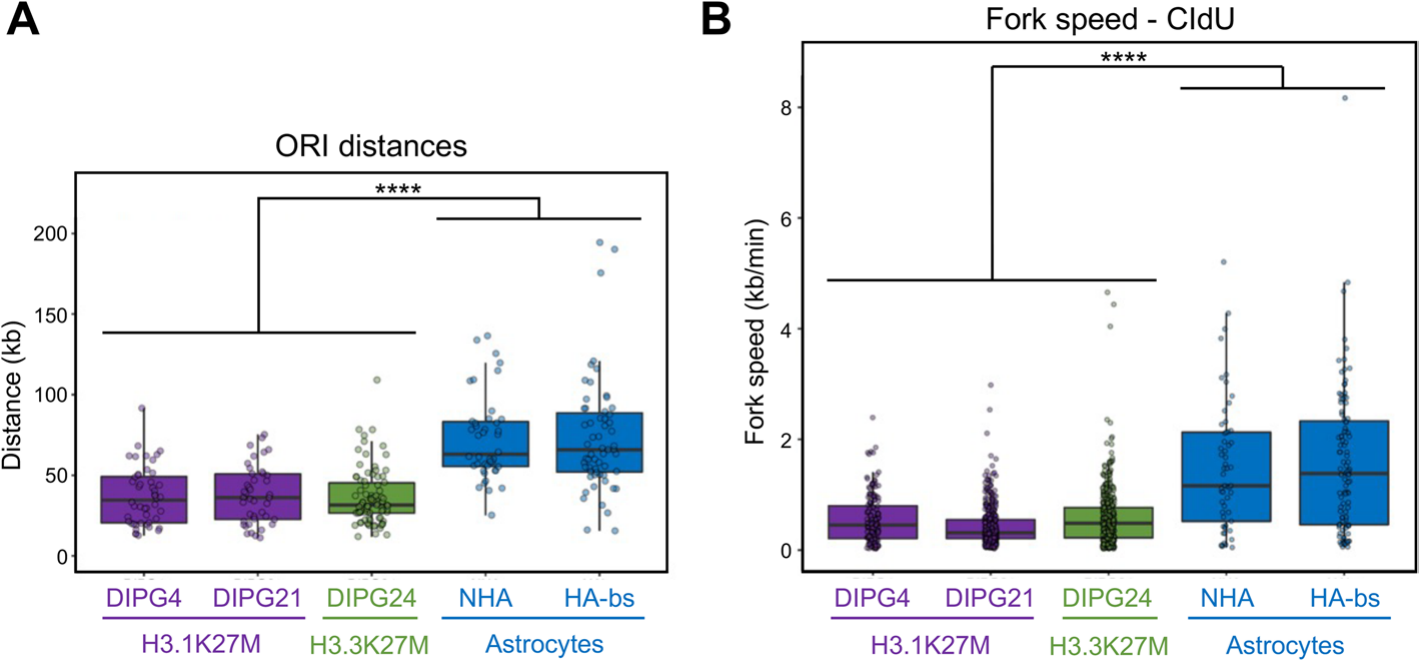
H3K27-altered DMG have more densely active origins of replication and slower replication fork speed.** A** Box plot representing the distance in kilobases between putative active origins of replication in H3.1K27M (purple), H3.3K27M (green), and astrocytes (blue). **B** Box plot representing DNA replication fork speed in kilobase per minute analyzed by chlorodeoxyuridine (CldU) labeling in H3.1K27M (purple), H3.3K27M (green), and astrocytes (blue). Box plots show the median and the interquartile range (IQR), and whiskers depict the smallest and largest values within 1.5 × IQR. Statistics: Wilcoxon’s *t* test: **** *p* < 0.0001

Because alteration of replication timing can be linked to changes in DNA replication forks [29], we then performed DNA fiber assays to determine potential alterations in replication fork dynamics genome wide. These assays revealed that DMG cells from both subgroups presented a significantly shorter inter-origin distance (ORI) than normal astrocytes (median of ~ 35 kb compared to ~ 65 kb, Fig. 5A), suggesting that more origins are fired in these cells during S phase. Furthermore, fork speed was significantly slower in DMG cells in comparison to normal astrocytes (median of ~ 0.5 kb/min compared to ~ 1 kb/min, Fig. 5B and Additional file 1: Fig. S5A).

Because slow replication fork speed is a hallmark/feature of replication stress [7], we anticipated that these cells would have increased DNA damage. Consistent with this notion, DMG cell lines from both subgroups presented phosphorylation of the histone variant H2A.X at Serine 139 when compared to normal astrocytes (gH2AX, Additional file 1: Fig. S5B).

Collectively, these data suggest that H3K27M promotes replication-associated cell cycle gene misregulation as well as replication timing alterations that are associated with replication stress.

### DMG tumors feature a replication stress signature

**Fig. 6:**
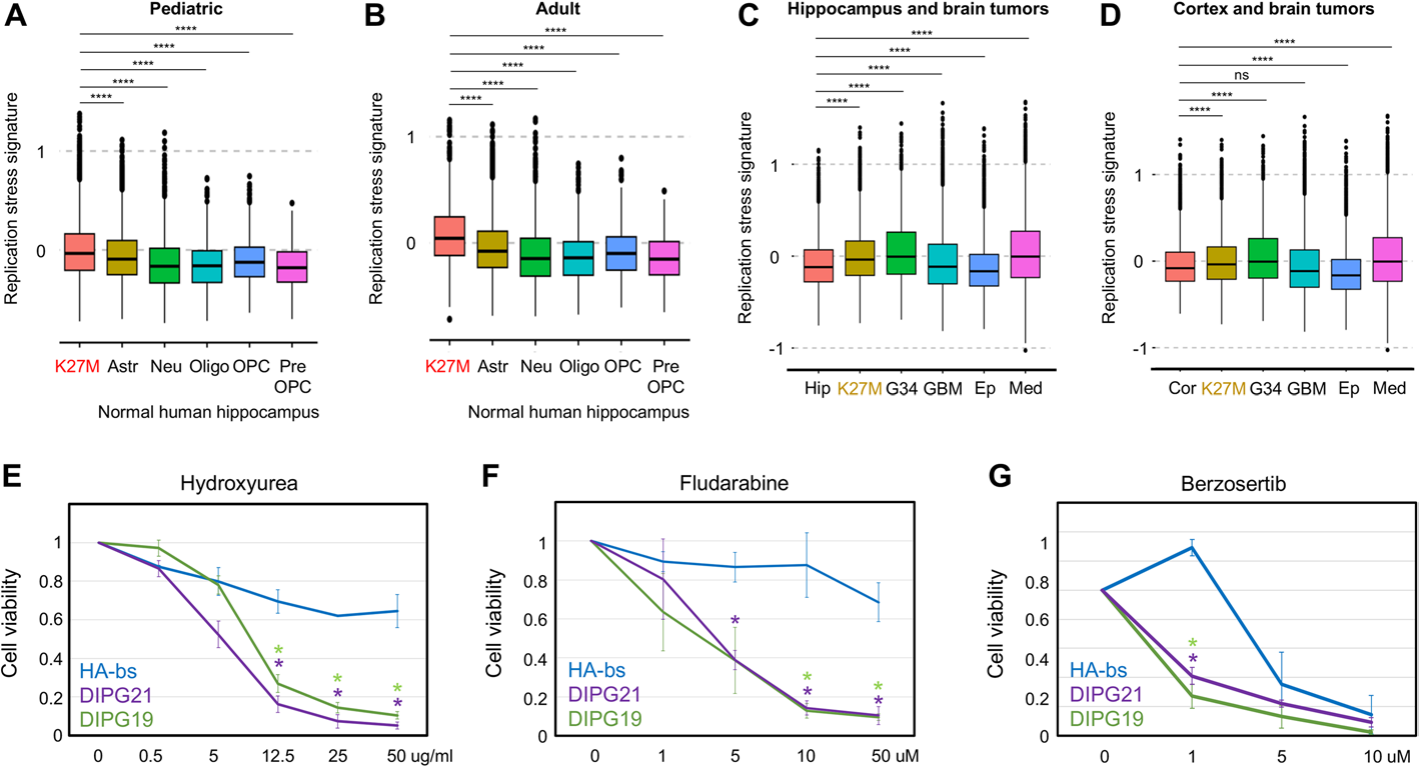
H3K27-altered DMG present replication stress and are vulnerable to replication stress therapy.** A** Replication stress signature score (as defined in [30]) in pediatric DMG tumors (*n* = 14) and normal human hippocampus (*n* = 6) from single-cell RNA sequencing. K27M = DMG, Astr = astrocytes, Neu = neurons, Oligo = oligodendrocytes, OPC = oligodendrocyte precursor cells, Pre-OPC = pre- oligodendrocyte precursor cells. **B** Replication stress signature in adult DMG tumors (*n* = 4) and normal hippocampus (*n* = 6) from single-cell RNA sequencing. K27M = DMG, Astr = astrocytes, Neu = neurons, Oligo = oligodendrocytes, OPC = oligodendrocyte precursor cells, Pre-OPC = pre- oligodendrocyte precursor cells. **C** Replication stress signature in normal hippocampus (Hip, *n* = 6), DMG tumors (K27M, *n* = 18), diffuse hemispheric glioma G34-mutant (G34, *n* = 9), glioblastoma (GBM, *n* = 35), ependymoma (Ep, *n* = 18), and medulloblastoma (Med, *n* = 36) from single-cell RNA sequencing. **D** Replication stress signature in normal cortex (Cor, *n* = 48), DMG tumors (K27M, *n* = 18), diffuse hemispheric glioma G34-mutant (G34, *n* = 9), glioblastoma (GBM, n = 35), ependymoma (Ep, *n* = 18), and medulloblastoma (Med, *n* = 36) from single-cell RNA sequencing. Statistics: adjusted *p*-value: ns *p* > 0.05, * *p* < 0.05, ** *p* < 0.01, *** *p* < 0.001, **** *p* < 0.0001. **E**–**G** Cell viability assays in the presence of the replication stress therapeutic hydroxyurea (**E**), fludarabine (**F**), and berzosertib (**G**) for 72 h (CellTiter-Glo 2.0 Cell Viability Assay, Promega). HA-bs (astrocytes), DIPG21 (H3.1K27M), and DIPG19 (H3.3K27M) are represented. Cell viability was calculated as a fraction of the control (water for HU and DMSO for fludarabine and berzosertib) viability. Statistics: Student’s *t* test: * *p* < 0.05 related to HA-bs

We then aimed to determine whether DMG tumors presented replication stress. We analyzed single-cell RNA sequencing data from DMG tumors and normal human hippocampus and assessed the presence of a replication stress response signature defined by the overlap between three key characteristics associated with replication stress (oncogene amplification, phospho-CHK1 protein expression, cell cycle checkpoint inhibitor response) [30]. Pediatric and adult tumors presented a higher replication stress signature than other cell types within the normal brain hippocampus (astrocytes, neurons, oligodendrocytes, oligodendrocyte precursor cells, pre-oligodendrocyte precursor cells; Fig. 6A–B).

We further explored whether this replication stress signature would be shared with additional brain tumors. Our comparison of normal hippocampus (Fig. 6C) or normal cortex (Fig. 6D) to DMG, diffuse hemispheric glioma G34-mutant, glioblastoma, ependymoma, and medulloblastoma demonstrates the replication stress signature to be present in oncohistone-driven brain tumors (DMG, G34) and medulloblastoma (Fig. 6C–D).

### DMG cells are susceptible to replication stress therapeutics in comparison to normal astrocytes

Based on the multi-omics data we generated in the cell models and linked to tumors, we assessed whether DMG cell lines could be sensitive to replication stress therapy. Specifically, we tested three clinically used compounds: the anti-metabolite hydroxyurea, which reduces the production of dNTPs, the purine analog fludarabine, which inhibits DNA synthesis through multiple mechanisms, and the ATR inhibitor berzosertib, which prevents DNA damage checkpoint activation. DMG cell lines were much more sensitive to all three compounds than astrocytes (Fig. 6E–G and Additional file 1: Fig. S6). Of note, the only DMG cell line that did not present increased sensitivity to replication stress therapeutics in comparison to normal astrocytes (Additional file 1: Fig. S6) presented very low levels of gH2AX in comparison to other DMG cell lines (Additional file 1: Fig. S5B). Together, these data established a pharmacologic vulnerability in DNA replication, which resolved a potentially novel therapeutic strategy for this non-curable disease.

## Discussion

Despite the recent advances in the understanding of the biology of DMG H3 K27-altered, these brain cancers are still not curable [31]. The K27M mutation is found in two histone genes: *HIST1H3B/C*, coding for the replicative histone H3.1, and *H3F3A*, coding for the histone variant H3.3 [11, 12]. These mutations define two DMG subgroups presenting differences in phenotypes, response to therapy, and prognosis [13].

In contrast to histone variants that are expressed throughout the cell cycle, replicative histones are expressed only during the S phase, so that newly synthesized DNA becomes properly chromatinized. H3.1 and H3.3 are enriched at specific places of the genome and have independent and complementary roles [14]. During DNA replication, old histones are recycled, and new histones are deposited. In the context of histone H3, the newly deposited histones are the replicative H3.1/2 [14]. Later during the S phase, H3.3 is repopulated at the regions it was enriched before replication [14]. This highly regulated deposition was recently shown to be important for proper DNA replication [14]. However, to the best of our knowledge, the consequences of the K27M oncogenic mutations in respective histones H3 variants on the timing of DNA replication had still to be determined.

Here, we demonstrate that DMG cell lines harboring the H3.1K27M or H3.3K27M mutations presented differential replication timing in comparison to astrocytes and between themselves. We further demonstrate that regions presenting differential replication timing also presented differential gene expression, with regions replicating earlier during the S phase increasing gene expression, and regions replicating later during the S phase decreasing gene expression. These results are consistent with active genomic compartments replicating early and inactive genomic compartments replicating late [2]. During development, different cell types harbor differential replication timing and gene expression [3]. The observation of differential replication timing for 10–12% of the genome in DMG cell lines compared to astrocytes may not be surprising, considering these to be different cell types [DMG being stalled in a cancer stem-cell-like state [17]]. However, a causal relationship between replication timing and gene expression has not been firmly established, and, in some instances, such relationships do not exist. For example, the differential replication timing due to the overexpression of a lysine demethylase in diploid cells did not correlate with changes in gene expression [1]. While further studies are necessary to determine potential causalities between replication timing and gene expression, a possibility would be for such relationships to exist when considering different cell types or when cell states are altered. Therefore, the fact that differential timing between H3.1K27M and H3.3K27M DMG cell lines correlated with differential gene expression could suggest these two DMG subgroups to be in different cell states and/or stalled in distinct differentiation stages. This would be consistent with recent findings highlighting the possibility of different cells of origin for these two subgroups [32].

Expression of genes involved in DNA replication, such as *ORC1* and *MCMs*, is tightly regulated across the cell cycle [33, 34]. However, a limitation of these previous studies is the use of drugs altering DNA replication to synchronize the cells, which could itself alter gene expression. Using cell sorting based on DNA content, we demonstrate that, in normal astrocytes, genes encoding the origin recognition complex, DNA replication licensing factors, DNA polymerase, and DNA repair machinery associated with DNA replication, presented increased expression between early S and late S phase. In DMG cell lines, these genes presented increased expression much earlier during the cell cycle, between G2 and G1 or between G1 and ES. Furthermore, these genes, especially cluster four (*ORC1*, *MCM2-3–4–6*), presented higher expression in DMG cells compared to astrocytes. ORC1 acts as a nucleating center for origin recognition complex assembly and then pre-replication complex assembly [35], having more ORC1 expressed earlier during the cell cycle could result in more origins firing, as observed in our assays, and in alteration in the timing of replication. The specific relationship between gene expression of DNA replication genes and alterations of DNA replication will need to be determined in future studies.

Increased replication initiation and/or origin firing can lead to the depletion of nucleotide pools, and, in turn, to slower fork speed and replication stress [7]. We noted the increased expression of thymidine kinase 1 (*TK1*) specifically in the early S phase of H3.1K27M DMG cells. TK1 is a cell cycle-regulated cytosolic enzyme from the DNA salvage pathway involved in regenerating thymidine for DNA synthesis [24, 25]. Future studies will need to determine whether this enzyme is a dependency for this subgroup of DMG. Of note, a genome-wide CRISPR screen identified de novo pyrimidine biosynthesis as a dependency in DMG cells. Inhibiting an enzyme from this pathway arrested the cell cycle by stalling replication forks, leading to replication stress and apoptosis in DMG cells but not in astrocytes [36]. Replication defects due to the overexpression of H3.3K27M were also reported in another study, revealing increased genomic instability upon replication stress [37]. Moreover, expression of H3.1K27M in fibroblasts decreased double-strand break repair induced by ionizing radiation and increased genome instability [38].

Standard of care therapy in DMG is radiation therapy, with a short-lived response. Replication stress therapy has the potential to increase sensitivity to radiotherapy. Hydroxyurea has been used as a radiosensitizer in combination therapies against cancers such as head and neck and cervix [39]. Furthermore, hydroxyurea, fludarabine, and berzosertib have the capacity to cross the blood–brain barrier, a limitation of therapeutic strategies in brain cancers. Future studies will need to assess whether such a combination could be beneficial in mice models of DMG.

## Conclusions

In this study, we demonstrated that, in comparison to normally dividing brain cells, DMG cell lines harboring the H3.1K27M or H3.3K27M mutations presented differential replication timing that correlated with differential gene expression. We further demonstrated that DNA replication-related genes were differentially regulated across the cell cycle and that DMG cell lines presented a smaller distance between the origin of replication and slower replication forks. Confirming findings in patient samples, DMG tumors presented a higher replication stress signature than the normal hippocampus. Finally, we uncovered a specific susceptibility of DMG cells to replication stress therapy. In summary, a comprehensive characterization of DNA replication timing in DMG and associated gene expression revealed targeting DNA replication as a potentially novel therapeutic strategy against DMG.

## Methods

### Cell culture

DMG cell lines were obtained from Michelle Monje (Stanford University). DMG cell lines were cultured in a base medium consisting of equal parts Neurobasal-A Medium (Thermofisher, 10,888,022) and DMEM/F-12 (Gibco, 11,330,032) to which were added 1% of the following: 1 M HEPES (Gibco, 15,630,106), GlutaMAX Supplement (Gibco, 35,050,061), 100 × MEM Non-Essential Amino Acids (Gibco, 11,140,050), 100 mM Sodium Pyruvate (Gibco, 11,360,070), and 100 × Antibiotic–Antimycotic (Gibco, 15,240,096). This base medium was supplemented with the following growth factors: 20 ng/ml EGF (STEMCELL, 78,006), 20 ng/ml FGFb (STEMCELL, 78,003), 10 ng/ml PDGF-AA (Shenandoah Biotechnology, 100-16AF-100UG), and 10 ng/ml PDGF-BB (Shenandoah Biotechnology, 100–18-100UG); and with 0.2% of B-27 Supplement Minus Vitamin A (50 ×) (Gibco, 1,287,010) and Heparin Solution (STEMCELL, 07980). Cells were passaged using the cell separator TrypLE Express (Gibco, 12,604,039) and Hanks’ Balanced Salt Solution (Corning, 21–022-CV) and resuspended in media as a single cell suspension. Cells were allowed to form neurospheres in preparation for experiments.

Normal human astrocytes (NHA) (Lonza, CC-2565) and Human Astrocytes-brain stem (HA-bs) (Sciencell, 1840) were purchased directly from the suppliers and cultured following the manufacturer’s instructions.

Cell lines were not authenticated after obtention. Cell lines were used at low passages and were free of mycoplasma contamination.

## Cell viability assays

Cells were seeded at 1 × 10^3^ density in triplicate in 96 well plates. After 24 h, media containing 2 × drug concentration was added in equal parts to media and cells in the plate. Hydroxyurea (Selleck Chemicals, S1896), fludarabine (MedChem Express, HY-B0069), and berzosertib (Selleck Chemicals, S7102) were incubated with cell lines for 72 h. Then, cell viability was measured using CellTiter-Glo 2.0 Cell Viability Assay (Promega, G9243) following the manufacturer’s instructions.

## DNA fiber assays

DNA fibers were prepared as described [29] based on [40]. Cells were sequentially pulse-labeled with 25 mM IdU (Sigma, I7125) and 50 mM CldU (Sigma, C6891) for 30 min each and harvested. Labeled cells were lysed by the lysis buffer (0.5% SDS in 200 mM Tris–HCl, pH7.4, 50 mM EDTA) and extracted DNA fibers were stretched onto the slide glass by tilting. The fibers were fixed in methanol/acetic acid (3:1), then denatured with 2.5 M HCl for 1 h, neutralized with PBS, and blocked with blocking buffer (1% BSA, 0.1% Tween 20 in PBS). CldU and IdU tracks were detected with anti-BrdU antibodies recognizing CldU (Abcam, Ab6326) and IdU (BD Biosciences, 347,580), respectively, and appropriate secondary antibodies (Thermo Fisher SCIENTIFIC, A11001 and A11077). Images were acquired on a Leica SP8 confocal microscope using a 40 × Plan/Apo NA1.3 oil immersion objective (Leica) at 2048 × 2048 pixels at an effective pixel size of 142 nm. Images were converted from Leica image format to TIFF files and fibers were annotated manually with a custom Fiji macro. From the saved fiber regions of interest, fiber patterns were automatically found in both CldU and IdU channels by a custom-build software written in Python (https://github.com/IES-HelmholtzZentrumMunchen/dna-fibers-analysis) followed by filtering for ongoing forks. Statistical analysis and preparation of plots was carried out in “R” version 4.1.2 with ggplot2 version 3.3.6. To calculate fork speed, we used the established conversion 1 mm = 2 kb [41]. To obtain the inter-origin distances, label boundaries were selected manually from annotated fiber regions aided by a custom Fiji macro. Origins were defined as the middle between two consecutive label boundaries along each fiber. All distances between neighboring origins were then used for statistical evaluation and plotting in “R,” using the same conversion factor as above.

## Replication timing sequencing

We adapted our replication timing sequencing protocol from two published protocols [18, 19]. Cells were incubated with 100 mM BrdU for 2 h, trypsinized, and filtered through a 100 mm Nylon mesh before being fixed with 100% EtOH. Cells were then resuspended in PBS/1%FBS with 1% Propidium Iodide and 0.25 mg/ml RNAse A and incubated for 1 h prior sorting. Cells were sorted in four equal fractions from S phase as described in Fig. 1A and in [18]. Two hundred thousand cells were collected per phase. Cells were then treated with proteinase K and DNA was extracted following the Zymo Quick-DNA Microprep Kit extraction instructions (Zymo Research, D3020). DNA was sonicated using a QSonica Q700 (10 min at 20% amplitude). Libraries were constructed using the NEBNext Ultra DNA Library Prep Kit (New England Biolabs, E7370L) as described in [19]. BrdU-labeled DNA was then immunoprecipitated and purified, followed by PCR amplification and purification as described in [19].

## Replication timing analysis

Repli-seq reads in each of the surveyed cell cycle phases (S1, S2, S3, S4) were mapped to the hg19 reference genome, followed by the removal of duplicates and counting reads over 50-kb bins across the genome. These counts were then quantile normalized and LOESS smoothed as described in [19].

## Replication timing index (RTI) and differential RT

The traditional metric of RT, early-to-late (E/L) ratio, is based on two time points (early and late S-phase). We generalized this metric to a more sensitive numerical value to quantify RT based on four time points in our experiments, or any other number of profiled timepoints *N*^3^ 2. The RT index (RTI) is based on a weighted sum of normalized replication signals (Repli-seq read densities *D*_*n*_) from each time point *n*:

$$RTI=\frac{{\sum }_{n=1}^{N}{n D}_{n}}{{\sum }_{n=1}^{N}{D}_{n}}$$

where *n* is the time point of the cell cycle (1 to 4, corresponding to time points S1 to S4) and *D*_*n*_ is the density of BrdU reads (per bp) within the given region at this time point. Regions of differential RT between cell types were identified using the difference of RTI in a given genomic bin, based on the cutoff of two standard deviations in pairwise comparisons of biological replicates, similar to [42, 43].

## Classification of local replication patterns

To identify types of local replication patterns, we analyzed RTI values at 50 kb genomic bins and surveyed RTI patterns over a window of 10 adjacent bins sliding across the genome. To represent the local shape of the RT pattern in each window, the RTI value in each of the 10 bins was normalized by the average RTI across window. The local patterns of constant RT, local RTI minimum, local RTI maximum, and RTI slope corresponded to constant timing regions (CTR, defined as a window with RTI variance < 0.006 among 10 bins), initiation zones (IZ, a local minimum of RTI), termination sites (TS, a local maximum of RTI) and the remaining genomic windows classified as timing transitioning regions (TTR), similar to [9]. We further subdivided the constant timing regions (CTRs) into initiation constant replication regions (ICTR) and termination constant replication regions (TCTR) based on RTI profiles in the flanking 500 kb windows.

## Cell cycle RNA sequencing

Cell cycle RNA-sequencing experiments were performed as described in [1]. Cells were incubated with Hoechst 33,342 (Invitrogen, H3570) at 1:3000 dilution for 1 h. Cells were then trypsinized, resuspended in Hoechst-containing media, filtered using a 100-mM filter, and sorted in four phases (250,000 cells per phase) as described in Fig. 1A in 1 mL of lysis buffer containing 10 mL of beta-mercapto-ethanol. RNA was then extracted following the PureLink RNA Mini kit (Invitrogen, 12,183,025) instructions with DNAse treatment (Invitrogen, 12,185,010). Libraries were prepped following the NEBNext Ultra II Directional RNA Library Prep Kit for Illumina instructions with rRNA depletion (NEB #E6310), followed by paired-end sequencing.

## RNA sequencing analysis

STAR aligner was used to map sequencing reads to transcripts in the hg19 reference genome [44]. Read counts for individual transcripts were produced with HTSeq-count [45], followed by the estimation of expression values using EdgeR [46]. Differential expression analysis was performed using EdgeR after normalizing read counts and including only genes with count per million reads (CPM) > 1 for one or more samples. Differentially expressed genes were defined based on the criteria of > twofold change in expression value and false discovery rate (FDR) < 0.05.

## Single-cell RNA sequencing

Single-cell RNA sequencing from DMG were performed in [47], from hippocampus in [48], from cortex in [49], from G34 in [50], from GBM in [51], from ependymoma in [52], and from medulloblastoma in [53].

## Western blot

Cell lines were lysed in RIPA. Lysates were sonicated in a QSonica Q800R for 10 min with 30 s “on” 30 s “off” intervals at 95% amplitude. Twenty-five milligrams of lysates were analyzed by western blot with antibodies against phospho-H2A.X(Ser139) (Sigma-Aldrich clone JBW301, #05–636) and b-actin (Abcam #8226).

## Supplementary Information

Additional file 1: this file contains supplementary figures S1 to S6, supplementary figures legends, and supplementary tables legends.

Additional file 2: this file contains supplementary tables S1 to S3.

Additional file 3: this file contains the uncropped blots of Figure S5B.

## Data Availability

The data generated in this study are publicly available in GEO under accession numbers GSE268775 and GSE268776 [54, 55]. Single cell RNA sequencing from DMG were previously published and are publicly available [47, 56], from hippocampus in [48, 57], from cortex in [49], from G34 in  [50,  58 ], from GBM in [51], [59], from ependymoma in [52, 60], and from medulloblastoma in [53, 61]. DNA fiber assays analyses were performed using a previously published and publicly available custom-build software written in Python (https://github.com/IES-HelmholtzZentrumMunchen/dna-fibers-analysis) [29, 62]. No other scripts and software were used other than those mentioned in the Methods section.
