# Supplementary material for: Multi-omics approaches reveal that diffuse midline gliomas present altered DNA replication and are susceptible to replication stress therapy
Source: Genome Biol. 2024 Dec 20;25:319. doi: 10.1186/s13059-024-03460-y (PMC11660928; doi:10.1186/s13059-024-03460-y)
Supplement: Supplementary file 1 — Additional file 1: this file contains supplementary figures S1 to S6, supplementary figures legends, and supplementary tables legends. [file 13059_2024_3460_MOESM1_ESM.docx]

**Supplementary figure and legends (Hains et al.)**


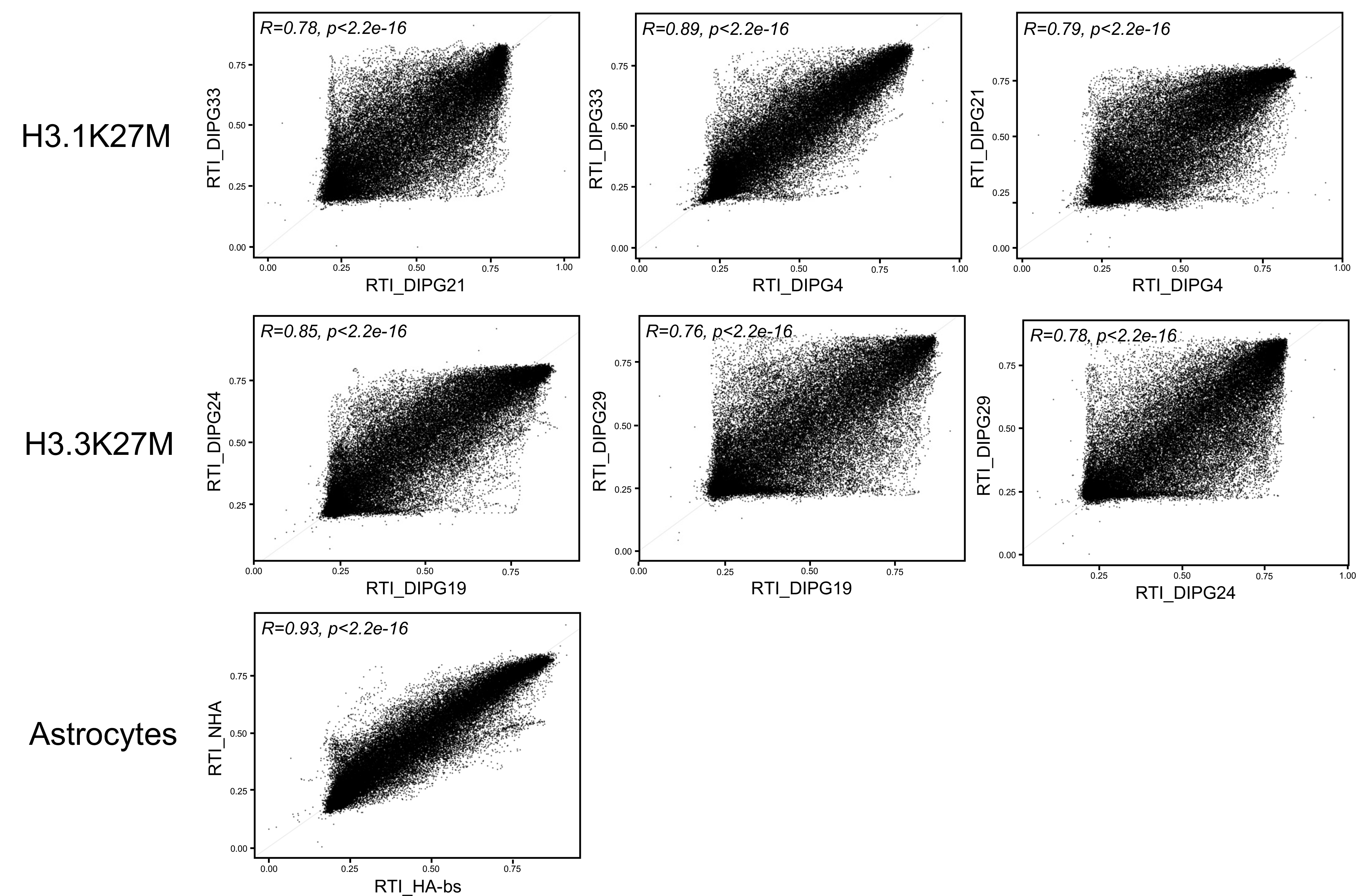


**Figure S1 (related to Figure 1).** Scatter plots comparing replication timing sequencing of cell lines within each subgroup [H3.1K27M mutation (DIPG4, DIPG21, DIPG33), H3.3K27M mutation (DIPG19, DIPG24, DIPG29), normal astrocytes (NHA, HA-bs)]. R = correlation coefficient.

**
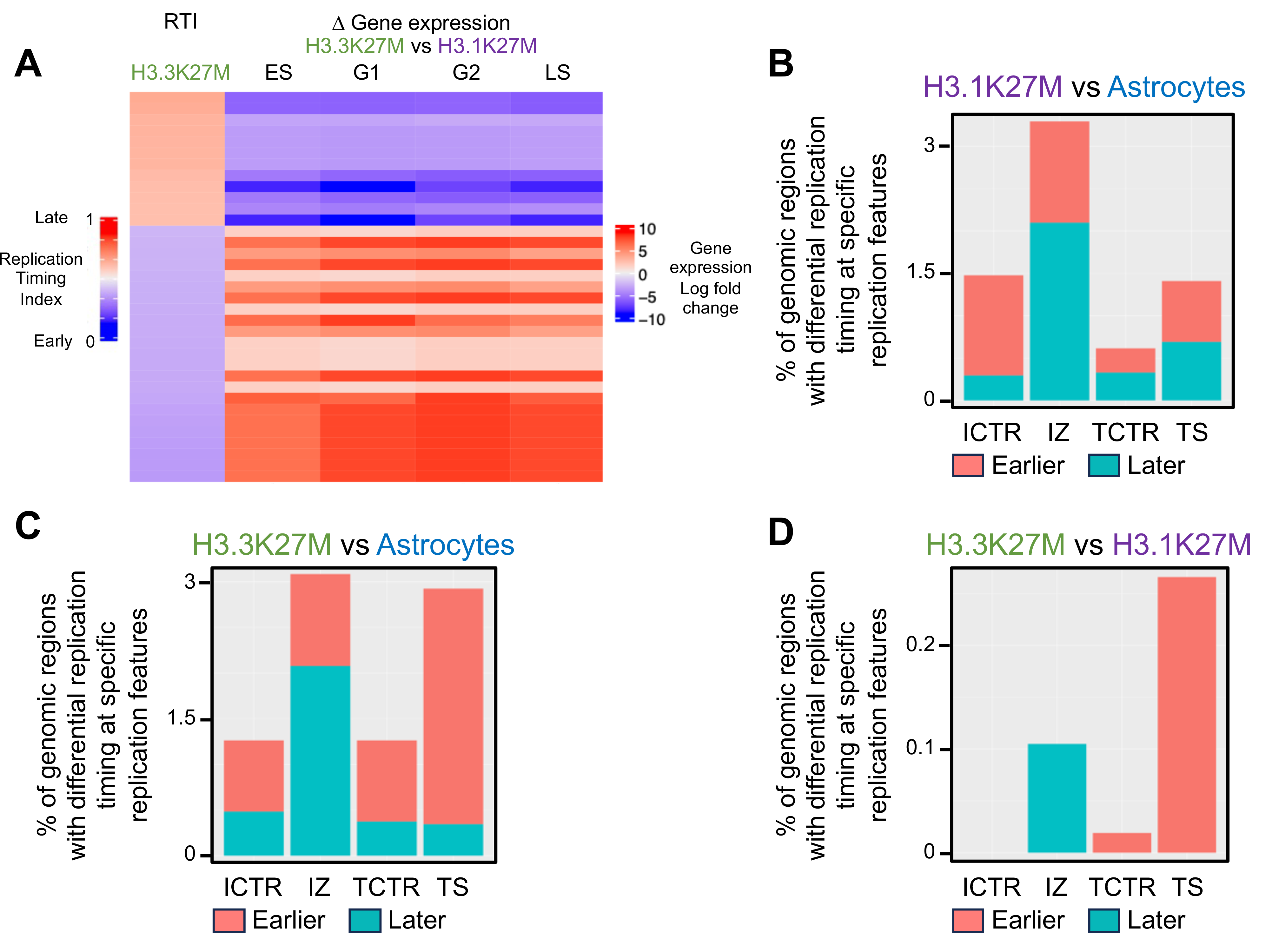
**

**Figure S2 (related to Figure 2). A.** Heatmap correlating the replication timing index (RTI, the value between 0 for early and 1 for late replication) with gene expression changes in the same regions (downregulated and upregulated in blue and red, respectively) for the genomic regions presenting differential replication timing between H3.3K27M and H3.1K27M DMG cell lines (35 regions). **B-D**. Differential replication timing at specific replication features of genomic regions presenting differences in RTI and in gene expression of H3.1K27M (**B**) and H3.3K27M (**C**) compared to astrocytes and of H3.3K27M compared to H3.1K27M (**D**) (earlier and later replication represented in coral and teal, respectively). IZ = initiation zones, TCTR = termination constant timing regions, ICTR = initiation constant timing regions, TS = termination sites.

**
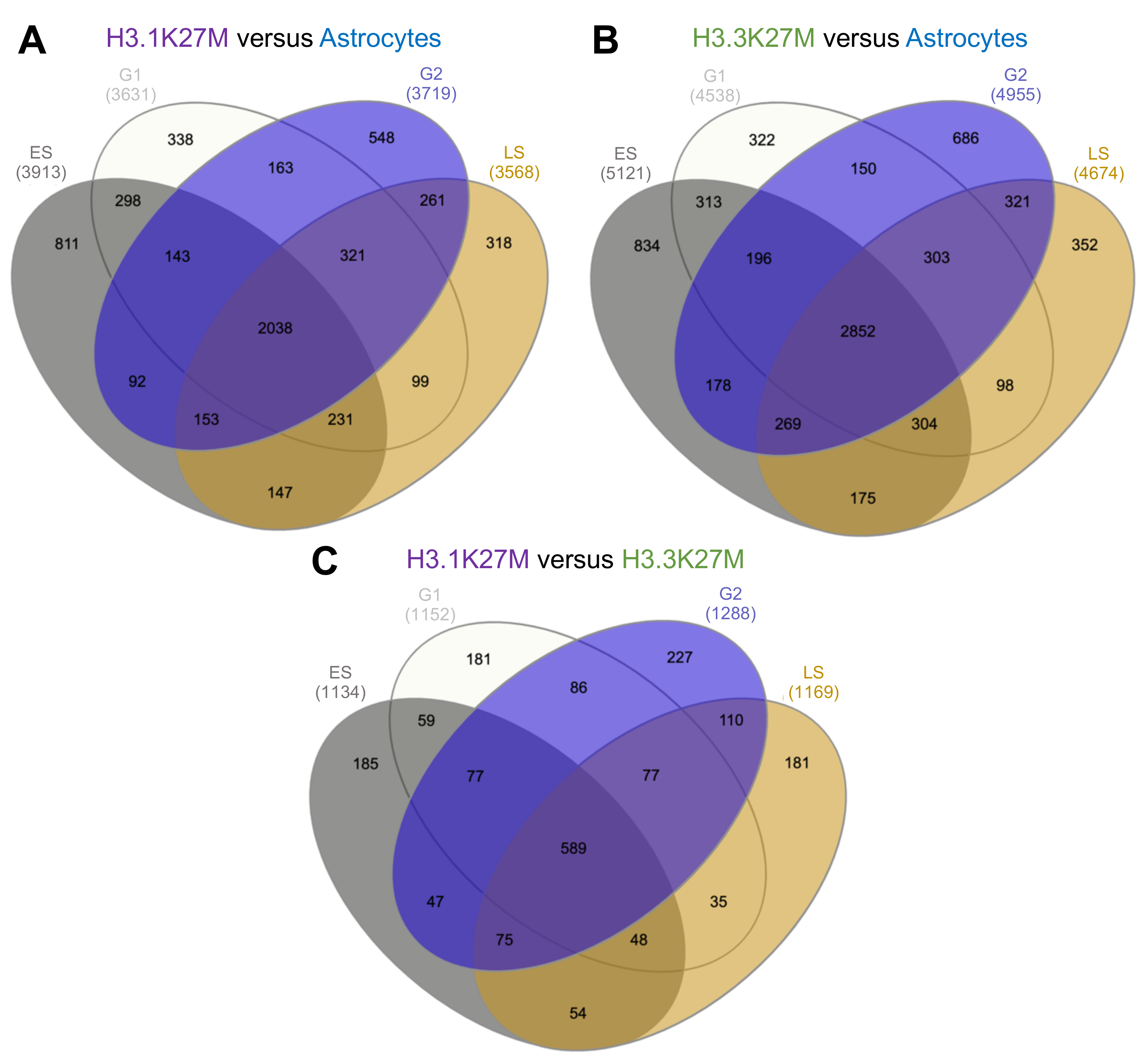
**

**Figure S3 (related to Figure 3).** Venn Diagrams representing the numbers of genes differentially expressed across four phases of the cell cycle (G1, ES = early S, LS = late S, G2) between H3.1K27M DMG cell lines and astrocytes (**A**), between H3.3K27M DMG cell lines and astrocytes (**B**), and between H3.1K27M and H3.3K27M DMG cell lines (**C**).


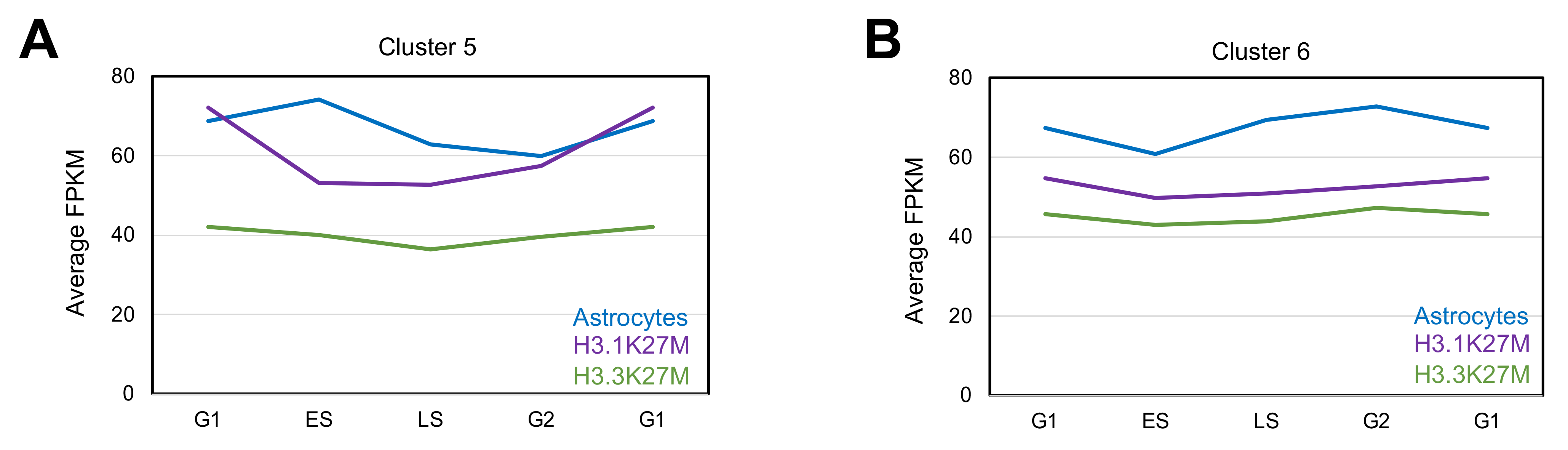


**Figure S4 (related to Figure 4). A-B.** Graphs representing the average of FPKM (fragments per kilobase per million mapped fragments) of genes from cluster 5 (**A**) and cluster 6 (**B**) for each phase of the cell cycle and each subgroup (astrocytes in blue, H3.1K27M in purple, H3.3K27M in green).


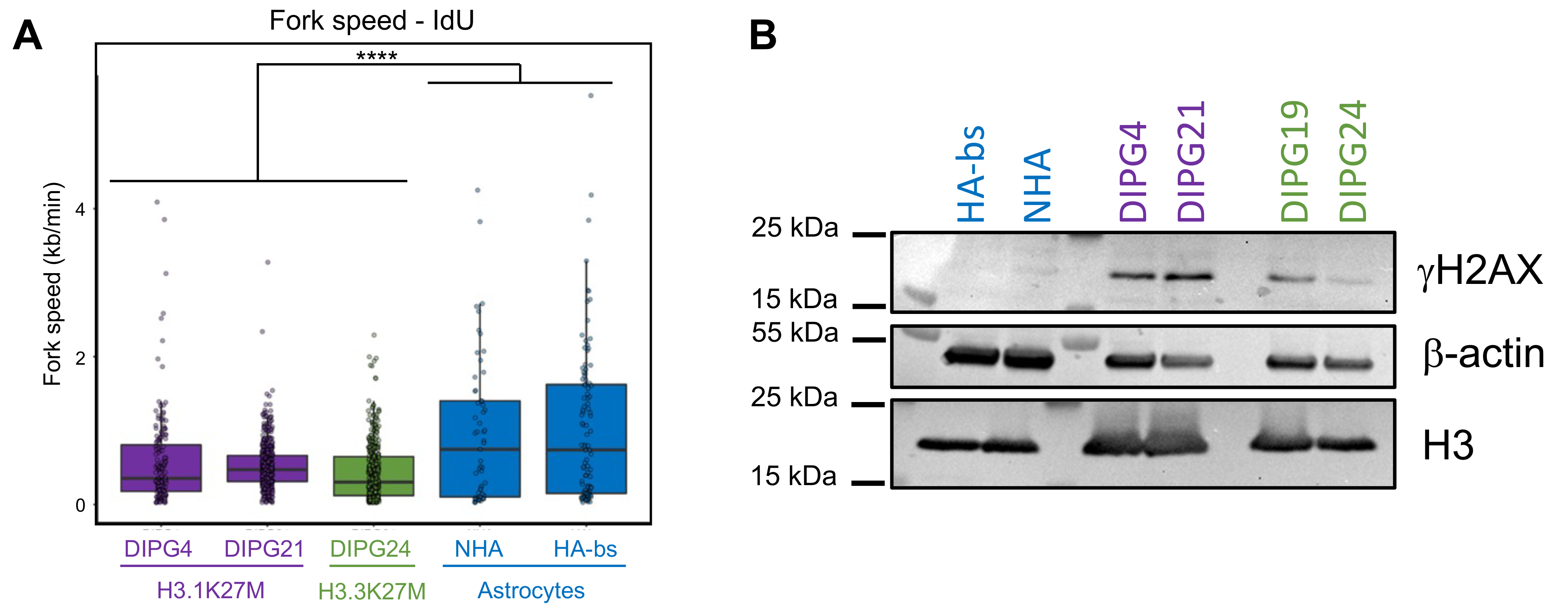


**Figure S5 (related to Figure 5). A.** Box plot representing DNA replication fork speed in kilobase per minute analyzed by iododeoxyuridine (ldU) labeling in H3.1K27M (purple), H3.3K27M (green), and astrocytes (blue). Box plots show median and the interquartile range (IQR), whiskers depict the smallest and largest values within 1.5 × IQR. Statistics: Wilcoxon’s t test: **** p<0.0001. **B.** Western blot representing phosphorylated-H2AX (γH2AX) in astrocytes (HA-bs, NHA) and DMG cell lines (H3.1K27M, DIPG4-DIPG21; H3.3K27M, DIPG19-DIPG24). β-actin and histone H3 were used as loading controls.


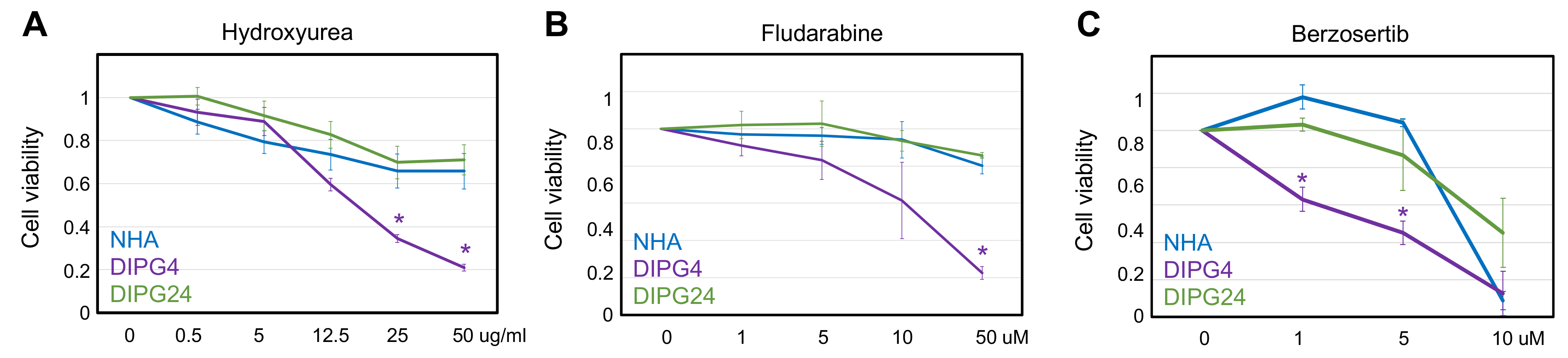


**Figure S6 (related to Figure 6).** **A-C.** Cell viability assays in the presence of the replication stress therapeutic hydroxyurea (**A**), fludarabine (**B**), berzosertib (**C**) for 72h in NHA (astrocytes), DIPG4 (H3.1K27M), and DIPG24 (H3.3K27M). Statistics: Student’s t test: * p<0.05 related to HA-bs or NHA.

**Table S1. Differential replication timing and gene expression of identical genomic regions in H3.1K27M compared to astrocytes.** The table reports genomic bins (column A) with their respective differential replication timing between H3.1K27M and astrocytes (column B), differential expression as log fold change in respective cell cycle phase (columns C to F) for each gene (column G). Replication timing indexes for each cell line are reported in columns H to L.

**Table S2. Differential replication timing and gene expression of identical genomic regions in H3.3K27M compared to astrocytes.** The table reports genomic bins (column A) with their respective differential replication timing between H3.3K27M and astrocytes (column B), differential expression as log fold change in respective cell cycle phase (columns C to F) for each gene (column G). Replication timing indexes for each cell line are reported in columns H to L.

**Table S3. Differential replication timing and gene expression of identical genomic regions in H3.3K27M compared to H3.1K27M.** The table reports genomic bins (column A) with their respective differential replication timing between H3.3K27M and H3.1K27M (column B), differential expression as log fold change in respective cell cycle phase (columns C to F) for each gene (column G). Replication timing indexes for each cell line are reported in columns H to L.
