# Supplementary figures and images for: Multi-omics approaches reveal that diffuse midline gliomas present altered DNA replication and are susceptible to replication stress therapy
Source: Genome Biol. 2024 Dec 20;25:319. doi: 10.1186/s13059-024-03460-y (PMC11660928; doi:10.1186/s13059-024-03460-y)

**Uncropped blots from Figure S5B (Hains et al.)**


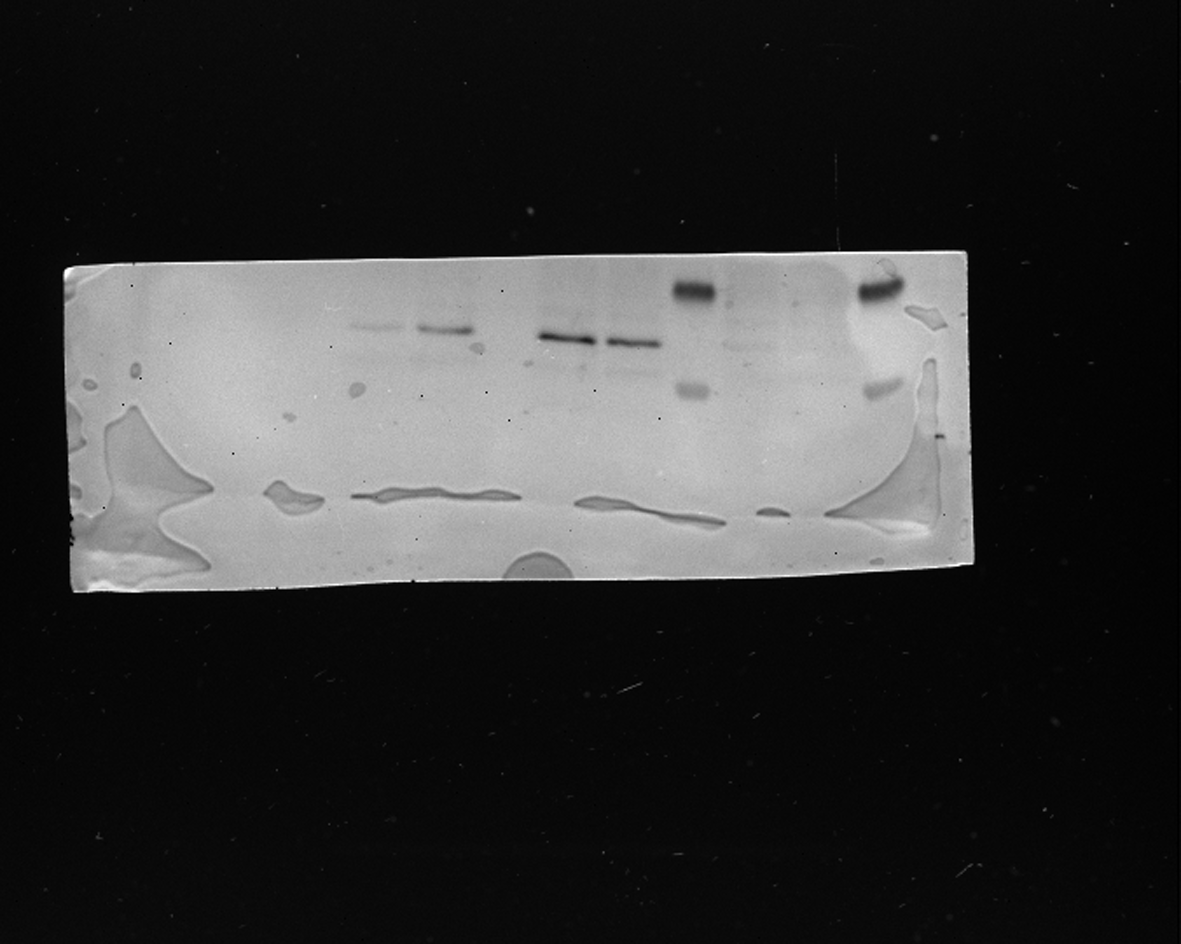


g-H2AX


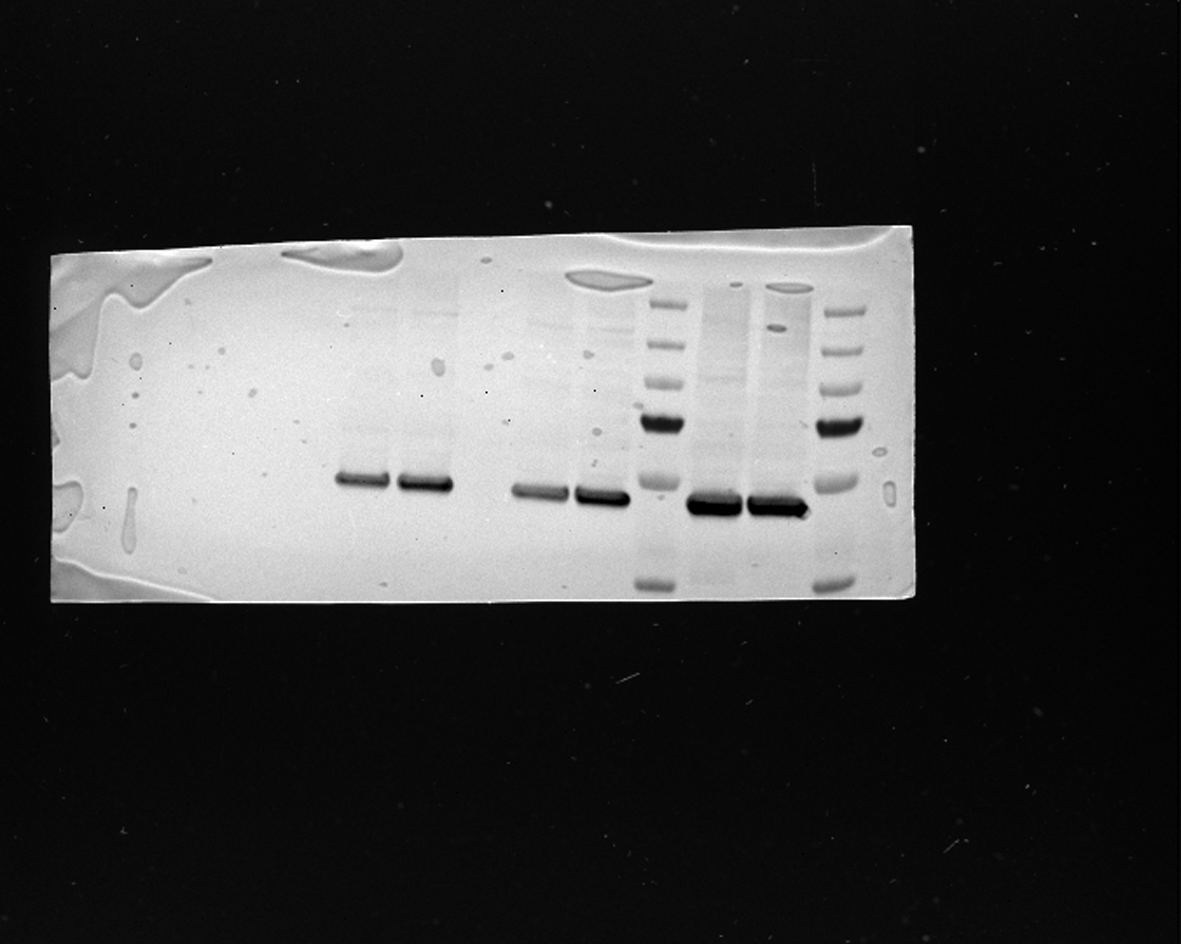


b-Actin


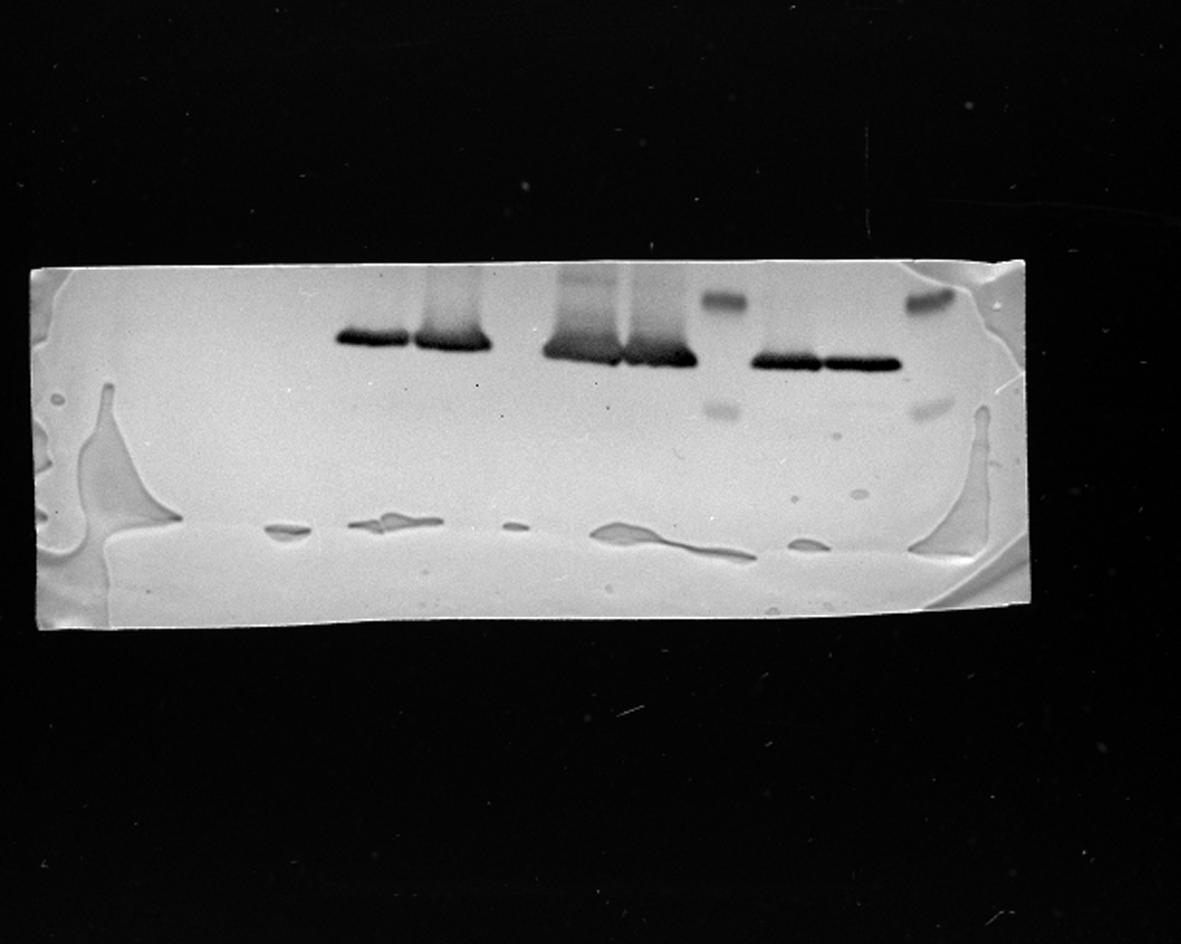


Histone H3

Supplement: Supplementary file 3 — Additional file 3: this file contains the uncropped blots of Figure S5B. [file 13059_2024_3460_MOESM3_ESM.docx]
